# Aesthetic Enhancement of the Brow using Hydroxyapatite

**DOI:** 10.1007/s00266-022-02793-y

**Published:** 2022-03-14

**Authors:** Lennert Minelli, Jacqueline Richa, Bryan C. Mendelson

**Affiliations:** 1 Melbourne Advanced Facial Anatomy Course (MAFAC), 109 Mathoura Road Toorak, Melbourne, VIC 3142 Australia; 2 The Panama Clinic, Calle Ramón H Jurado, Centro Pacific Center Torre B, Panama, Panama; 3 The Centre for Facial Plastic Surgery, 109 Mathoura Road Toorak, Melbourne, VIC 3142 Australia

**Keywords:** Hydroxyapatite, Orbit, Orbital rim, Brow, Temple, Skeletal augmentation, Volumizing

## Abstract

**Background:**

An aesthetically pleasing appearance of the ‘eyes’ usually includes good projection of the outer brow. Weak bony projection of the superolateral periorbital region tends to be not only less attractive, but also predisposes to hooding over the temporal part of the upper lid. Congenital lack of skeletal volume is exacerbated by ageing due to lipoatrophy and soft tissue laxity. The rationale and technique for performing skeletal augmentation of the superolateral orbital rim is described, along with long-term results from a series of cases.

**Material and Methods:**

A series of patients having augmentation of the superolateral orbital rim, using the technique described, were evaluated. A forehead crease incision was used, then a precise subperiosteal pocket developed in the lateral brow region between the supraorbital foramen and the superior temporal septum. The hydroxyapatite granule mixture was incrementally placed using modified syringes. The patients were followed to assess the long-term results.

**Results:**

Two hundred and fifty patients, 80% women, mean age = 53 years [range 23–78] underwent supraorbital rim augmentation using subperiosteal hydroxyapatite granules, during a 12-year period, commencing in 2007. The mean follow-up was 41 months (range 1–12 years). The mean volume used for augmentation was 1.0 mL per side (range 0.4–2.3 mL). Projection of the upper lateral periorbital prominence was effectively increased, resulting in enhancement of the brow position and shape. Twenty-seven patients (11%) had an undercorrection, requiring additional volume augmentation, all during the first three years of the experience. Twelve patients (5%) required correction of contour irregularities. There were no infections and no long-term complications. Resorption of the hydroxyapatite volume over time was not noted.

**Conclusion:**

The aesthetic significance of superolateral orbital rim projection is introduced. Patients who have a degree of skeletal deficiency of the zygomatic process of the frontal bone should be considered for hydroxyapatite augmentation of the bone as a complement to upper lid blepharoplasty and brow elevation. This procedure should be considered in the spectrum of upper periorbital aesthetic procedures.

**Level of Evidence IV:**

This journal requires that authors assign a level of evidence to each article. For a full description of these Evidence-Based Medicine ratings, please refer to the Table of Contents or the online Instructions to Authors www.springer.com/00266.

**Supplementary Information:**

The online version contains supplementary material available at 10.1007/s00266-022-02793-y.

## Introduction

*There cannot be a more important subject for the observation of the artist, than the form of the frontal bone. Much of the character of the whole head will be found to depend on the contour of the forehead, the ridges of the temples, the prominences formed by the cavities in this bone, and lastly, the arch of the orbit.*

*- Sir. Charles Bell, Essays on the Anatomy of Expression in Painting. London 1806*

**Fig. 1:**
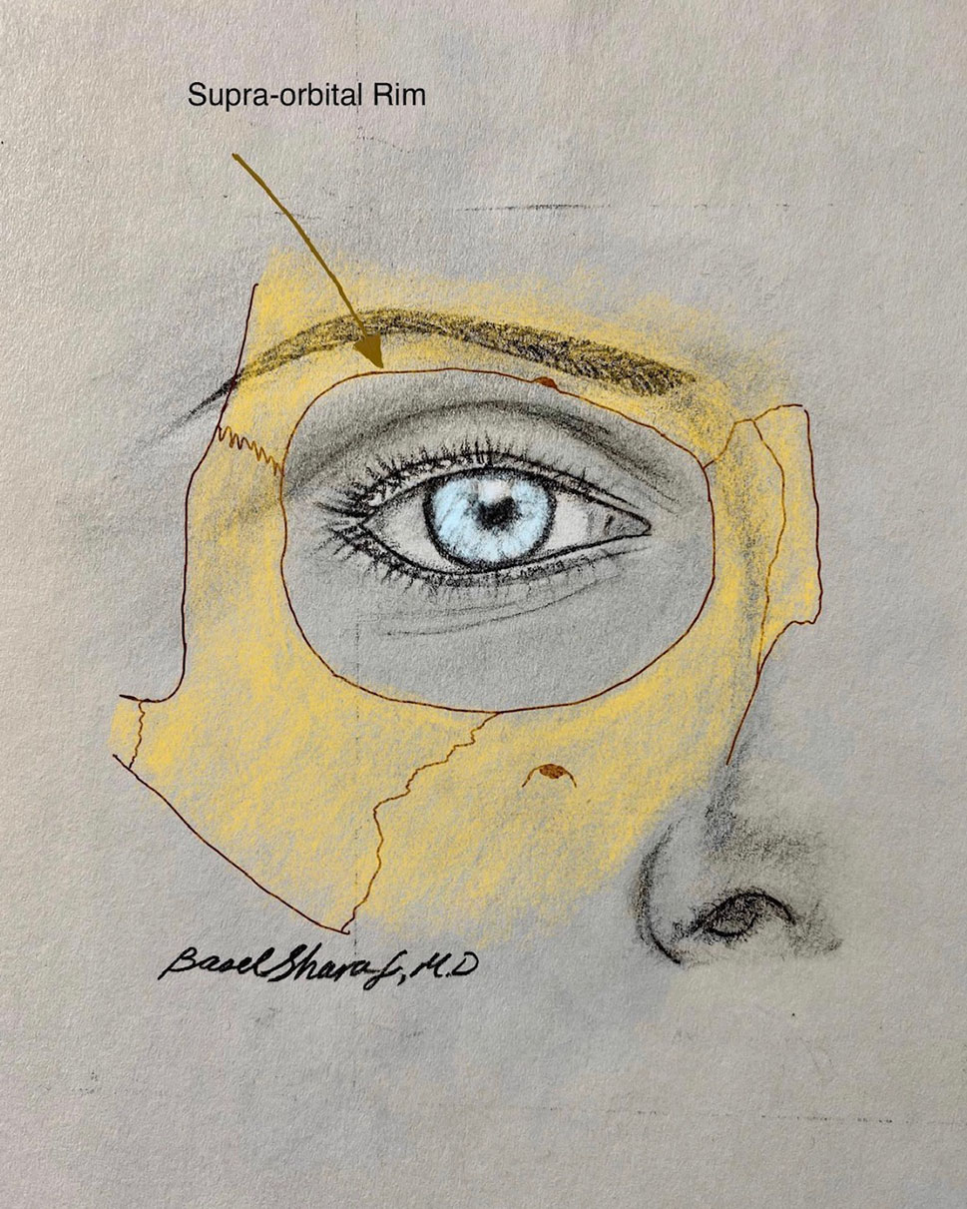
Artist rendering of the periorbital aesthetic unit. The skeletal projection of the lateral end of the superior orbital rim, formed by the zygomatic process of the frontal bone influences the shape, peak and position of the eyebrow curvature

**Fig. 2:**
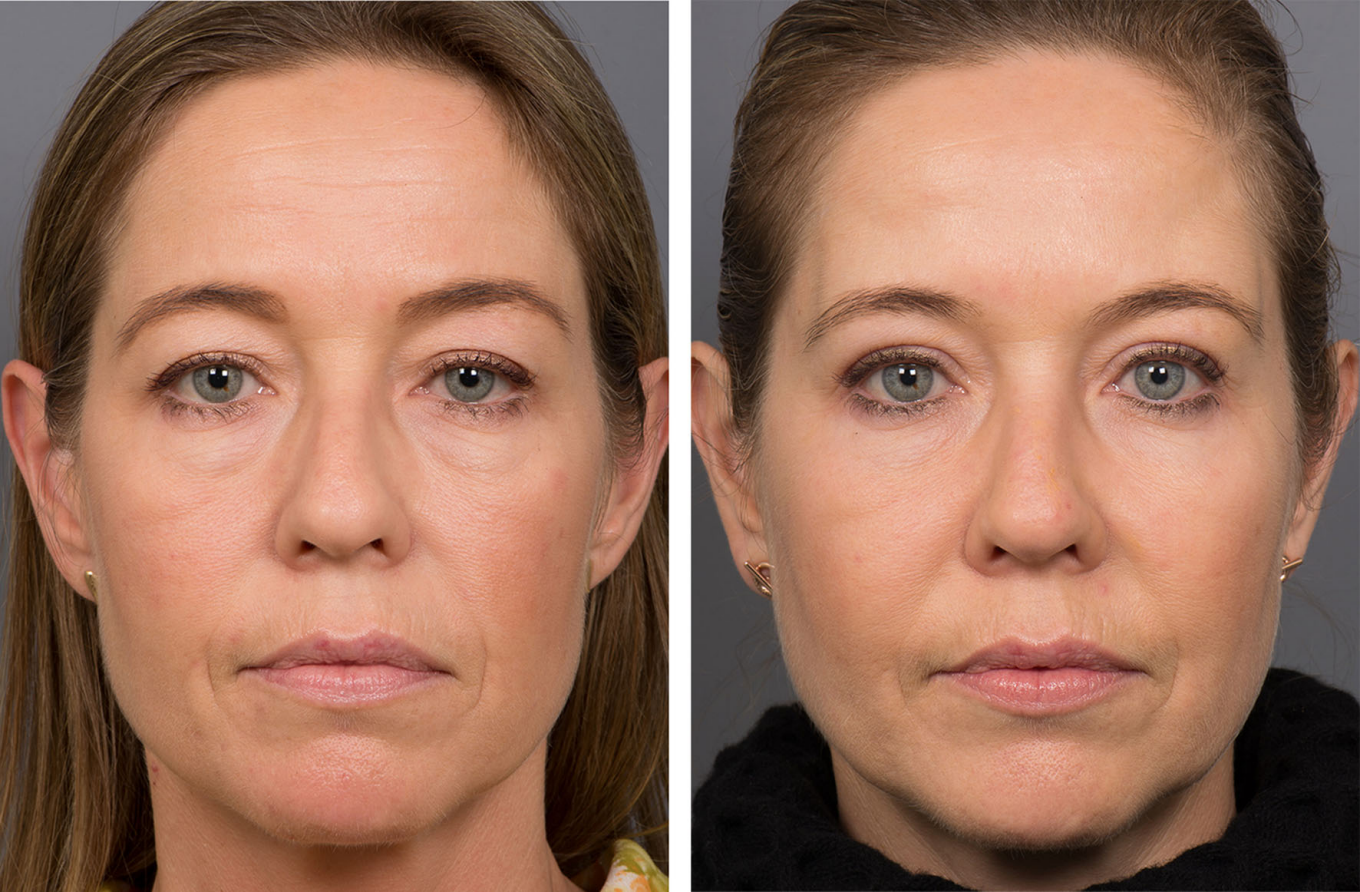
This case demonstrates a key principle. The perception of an improvement of the facial appearance overall, that results from a small improvement in the hierarchically important upper periorbital region. Left: This woman, age 43 requested a discrete improvement of her ‘tired look’, but not to be detectably obvious surgical. Surgery involved a combination of tarsal fixation upper lid blepharoplasty with conservative skin excision, and superolateral orbital rim augmentation using a hydroxyapatite mixture, 1.0 mL. For balance, a subtle midcheek improvement, transconjunctival lower lid fat adjustment and small onlay augmentation on the zygoma and maxilla was performed. No other surgery or filler. Right: One year postop. The improved definition between the lateral forehead and temple region visually broadens the periorbital part of the forehead above the brow, which in turn gives the sense of a more attractively defined face. While the better supported outer brow ‘opens’ the heaviness of the lateral part of the eyes. ‘A small change in facial proportion changes our perception of a persons’ personality’ Egon Brunswik 1934. Viennese Psychologist

The upper periorbital region is inherently an area of focus in facial aesthetics related to the central role of the ‘eyes’ in interpersonal communication and facial expression [1]. The upper periorbital region includes the eyebrows and upper eyelids, bounded superiorly by the forehead and laterally by the temple. These components have an intricate interdependent relationship and, as such, should be evaluated and treated as a unit, as seen in the artist’s rendering of the periorbital aesthetic unit (Fig. 1) [2]. Given it is such a hierarchically significant region of the face, small variations of the periorbital framework may have a significant impact on the perception of overall facial appearance, including age, personality, and expression (Fig. 2) [3, 4]. The concept of visual illusion is not generally thought of in surgery, but illusion impacts on nuances of proportion in periorbital aesthetics [4].

**Fig. 3:**
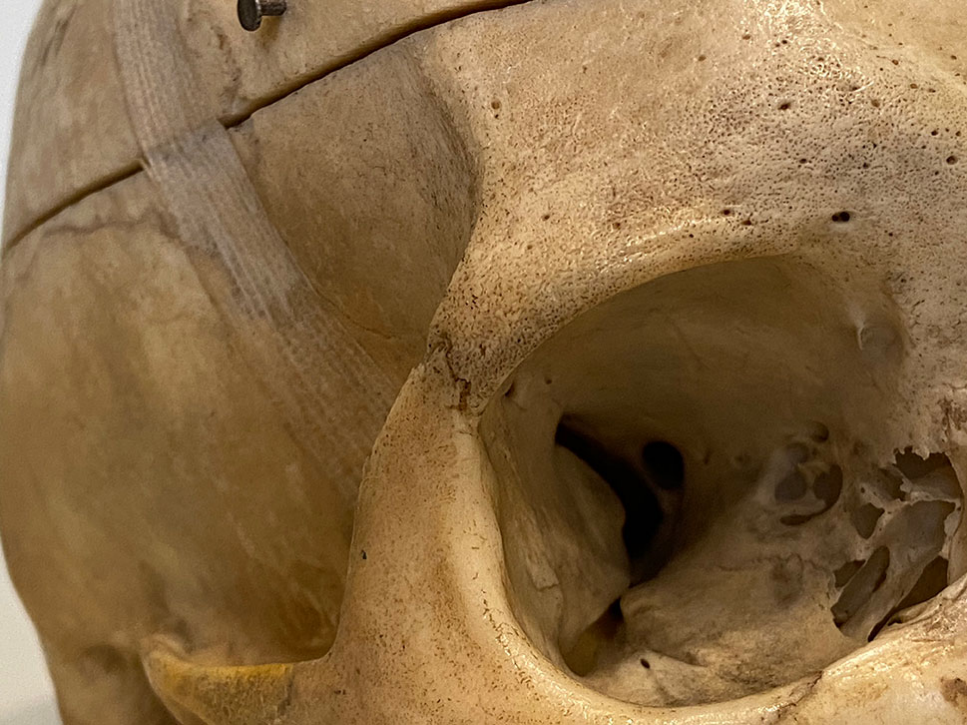
The maximum projection, forward and lateral, of the process, is the angulation above the suture line between the zygomatic process of the frontal bone and the frontal process of the zygoma

Brow and forehead enhancement has been guided by the notion of an aesthetic ideal [3, 5–10]. A broad forehead tends to be squarer and with good forward projection of the frontal bone is innately perceived as attractive. The lateral brow should have an appropriate fullness where it transitions into the adjacent temple aesthetic unit [11]. This transition is defined anatomically by the lateral promontory of the frontal bone, which here is the zygomatic process of the frontal bone. Even small variations in the prominence of the zygomatic process impact on the look of this region (Fig. 3) [12]. Individuals in whom the zygomatic process is larger feature a broader forehead with better brow support (Video 1). Accordingly, the eyebrows, which begin medially, at or about the level of the brow ridge, reside on the ridge itself in its middle one-third, and end laterally above the ridge [13]. In contrast, if projection of the lateral orbital rim is inadequate the lateral brow tends to sit on the inferior edge of the lateral brow ridge (Fig. 3). This causes the soft tissues to roll into the orbital aperture, with the appearance of brow descent and even lateral orbital hooding [14, 15]. The superior orbital rim has been described as receding with age, resulting in blunting of the ridge and an overall reduced prominence [15–18]. However, it has recently been demonstrated that the orbital aperture remains essentially stable throughout a lifetime [19]. Instead, it is the inherent, skeletal projection of the individual, which is the primary factor. If the projection is relatively deficient, ageing in the form of lipoatrophy and soft tissue ptosis in the upper periorbital region contributes to further deprojection with progressive descent of the brow [20–24].

Lifting of the brow in the presence of infrabrow volume deficiency can inadvertently magnify the volume loss appearance by further emphasizing the deepening of the supratarsal sulcus [25, 26]. Conversely, restoring the soft tissue volume of the upper eyelid and brow can reduce the need for a separate browlift [27, 28]. However, an excessive orbital aperture even in the presence of adequate soft tissue volume can induce superior hollows [29]. The adoption of refined lipofilling has provided an important advance in periorbital surgery. Separate from that, surgical augmentation to improve facial bone structure has been shown to significantly enhance a person’s appearance by providing a more attractive and youthful look [30–34]. This paper is intended to provide the reader with a clear understanding of the aesthetic benefits of skeletal augmentation of the periorbital region, and the ease of the procedure. To this end, our significant experience with the use of hydroxyapatite granules for augmentation of the superolateral orbital rim is presented.

## Materials and Methods

### Surgical Technique

Porous hydroxyapatite granules were used in a standard mixture, consisting of an approximately equal volume of the granules with the patient’s blood (drawn from a peripheral line), along with a haemostatic agent, initially collagen (Avitene™, Bard, New Jersey, USA) or EACA powder (Spongostan™ Powder, Johnson & Johnson, Somerville, NJ. USA) as previously described [30, 35–37]. Coral-derived hydroxyapatite granules were used. From the beginning of the series, these were Interpore 200™ (Interpore Cross International, Irvine, CA) later renamed Pro Osteon 200™ Biomet, until this product was discontinued in 2018. Afterwards, we changed to the allograft cortico-cancellous human bone chips Oravance™ (Australian Biotechnologies, NSW, Australia). Currently we are using a new generation hydroxyapatite, InRoad (which is a synthetic, enhanced version of the coral hydroxyapatite) although these cases are not included in this reported series. The mixture is packed into modified 1cc syringes, of which the hub end is cut off at a bevel, as the mixture is too coarse to pass through the standard hub.

All but a few of the augmentation procedures were performed under general anaesthesia. Intravenous antibiotic coverage (Cefazolin) was given with the induction. The area of intended augmentation is marked on the skin as well as off-limit areas. The superior temporal bony line, the location of the superior orbital rim and the supraorbital notch are marked. The patient is requested to elevate the brow, so a significant crease can be selected and marked for the incision. Local anaesthetic infiltration of the area is performed (8 to 10 mL xylocaine and adrenaline) to obtain near complete haemostasis as well as analgesia.

**Fig. 4:**
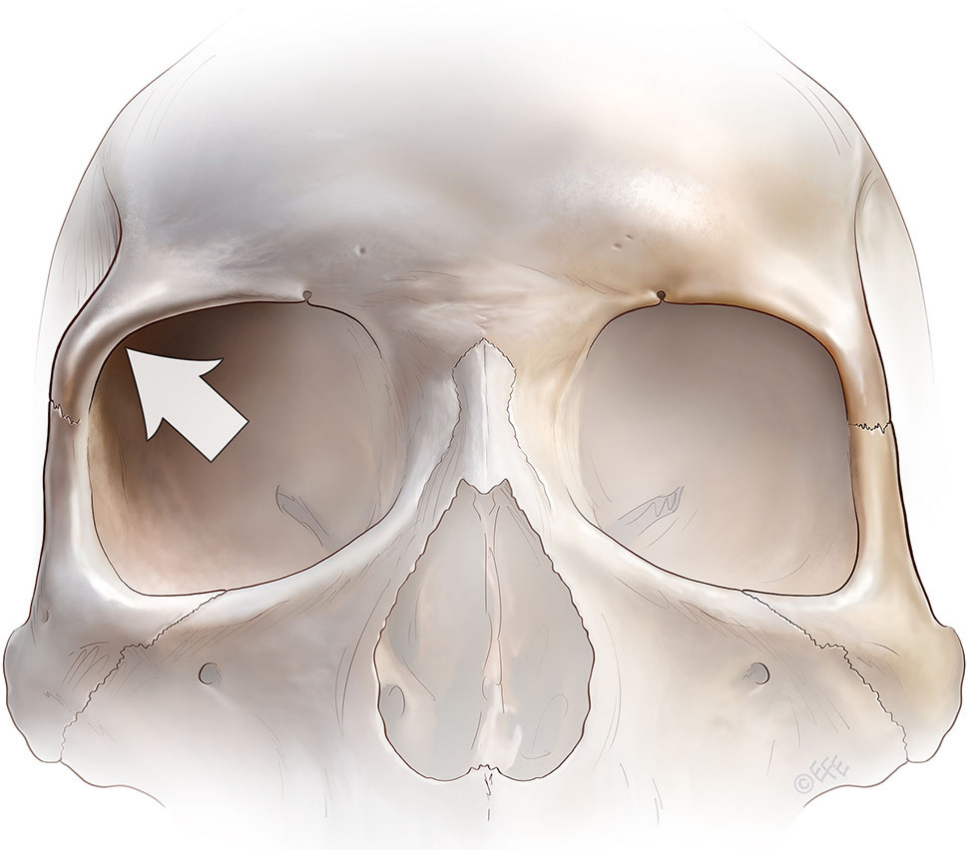
The arrow highlights the effect of a strong zygomatic process on the brow position on the right side. Whereas the weak projection of the left superolateral orbital rim does not provide good support for the brow, which then becomes ptotic and appears weak. The projection of the right zygomatic process supports the overlying soft tissues, which results in a strong and high-peaked brow with a more ‘open eye’ appearance, while the increased lateral projection broadens the lower forehead

A short transverse skin incision (12 to 15 mm) is placed in the premarked forehead crease. The periosteum is then incised approximately 5 mm caudal to the skin incision, to provide a flap valve to ensure soft tissue coverage at the conclusion of the procedure (Fig. 4a). A precise subperiosteal pocket is then gently elevated using a narrow curved periosteal dissector. The resulting triangular-shaped area has the following extents: inferiorly just over the lower edge of the supraorbital rim curvature, being careful to not over-dissect and lose control of the implant shape; medially to the vicinity of the supraorbital notch; laterally to the edge of the zygomatic process of the frontal bone and the superior temporal septum, being careful not to enter the temporal compartment (Fig. 4a) [38].

The hydroxyapatite mixture is placed along the superior orbital rim in a stepwise sequence using 0.2 mL increments until the desired volume of augmentation is achieved, rather than the routine overcorrection previously described by Byrd [30]. Precise digital moulding, aided by the periosteal elevator in the cavity and compaction of the granules allows for the creation of the intended contour (Fig. 4c–e; Video 2).

The periosteal pocket does not require suture closure as the entry opening effectively functions as a flap valve in conjunction with the skin incision closure and a local compression dressing. Prior to wound closure, suction cleansing ensures no residual granules remain in the soft tissues outside the actual implant area. Minor contour irregularities in the augmented region can be smoothed with firm manual pressure. Postoperative splinting with adhesive tape (Steri-Strip™, 3M, St. Paul, Minnesota) is placed over the temple and left in place for several days.

If concurrent autologous fat grafting is planned, this is usually performed as a first step prior to the creation of the subperiosteal pocket, to preclude breaching the periosteal envelope. Any autologous fat inserted is in the supraperiosteal, retro-orbicularis oculi fat (ROOF) and subcutaneous planes.

### Data Analysis

A retrospective review was undertaken of all patients who had undergone skeletal augmentation of the superolateral orbital rim using porous hydroxyapatite granules performed by the senior surgeon (BCM) over a 12-year period (December 2007 to March 2020) in March 2021. This included early cases during the necessary surgical learning curve. Recorded variables included age, gender, presence of comorbidities, smoking, previous aesthetic facial procedures, volume of the hydroxyapatite used at each site, incision used for surgical access, other locations of hydroxyapatite augmentation simultaneously performed, simultaneous procedures performed (including autologous fat grafting to the brow region), complications, revision procedures and duration of clinical follow-up.

## Results

Two hundred and fifty patients underwent skeletal augmentation of the superolateral orbital rim for aesthetic enhancement using porous hydroxyapatite granules, in the period from December 2007 to March 2020. The mean operation time was 15 min per side. Thirteen patients were excluded from the definitive analysis due to incomplete data. In total, 237 patients who received 245 surgical procedures for hydroxyapatite augmentation of the superolateral orbital rim were analysed.

### Patient Demographics

The mean patient age was 52 years (range = 23–78 years), most patients were female (*n* = 189, 80%). Most of the patients were non-smokers (*n* = 214; 90%) and had no comorbid conditions (*n* = 164, 69%). In those who had comorbidities, the most common were hypertension (*n* = 28), arthritis (*n* = 15) and hypothyroidism (*n* = 10). There were only six diabetic patients. Most of the patients had undergone one or more aesthetic facial surgical procedures performed in the past (*n *= 148, 62%). The mean follow-up was 41 months (range = 1–12 years; median = 24 months).

### Surgical Procedure Data

The average volume of hydroxyapatite granule mixture placed in each temple region was 1.0 mL (range = 0.4–2.3 mL; mean = 0.96 mL). Most patients had the same volume of hydroxyapatite placed on both sides, although in 19 patients slightly different volumes were placed to compensate for minor asymmetries of their bony anatomy.

Ageing lipoatrophy in the upper periorbital region was simultaneously addressed in more than half the cases (n = 143; 60%) with autologous fat grafting. The patients receiving autologous fat grafting were on average older (53-year-old vs 49-year-old, *P* value 0.01). Most patients (*n* = 176; 74%) had facial skeletal augmentation performed in multiple sites, which included: maxilla, zygoma, supramental groove, prejowl, mandibular rim, glabella, and temple. Most patients underwent other facial aesthetic procedures at the time of the supraorbital rim augmentation, not limited to blepharoplasty, temporal lift, concentric malar lift, and facelift.

### Operative Results

**Fig. 5:**
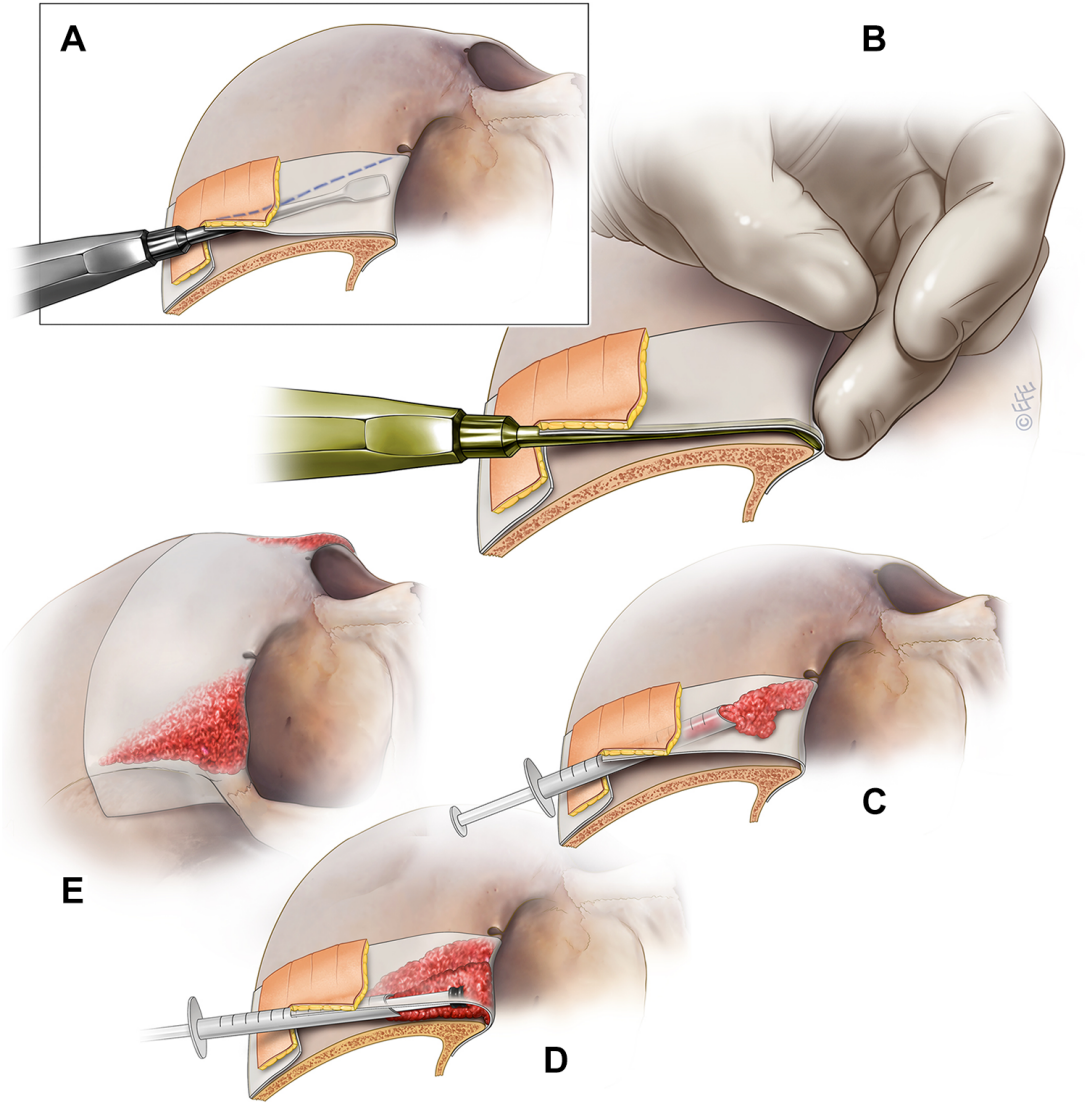
Steps in the surgical procedure.** a** Initial subperiosteal dissection medially. **b** To avoid over-dissection, a ‘curved on the flat’ dissector is used with finger palpation of the rim. **c** The first syringe volume placed medially. **d** Additional aliquots placed progressively further lateral. The plunger is used to position and shape the implant volume. **e** Final implant with the volumes increasing laterally and tapering up the periosteal boundary along the superior temporal line

**Fig. 6:**
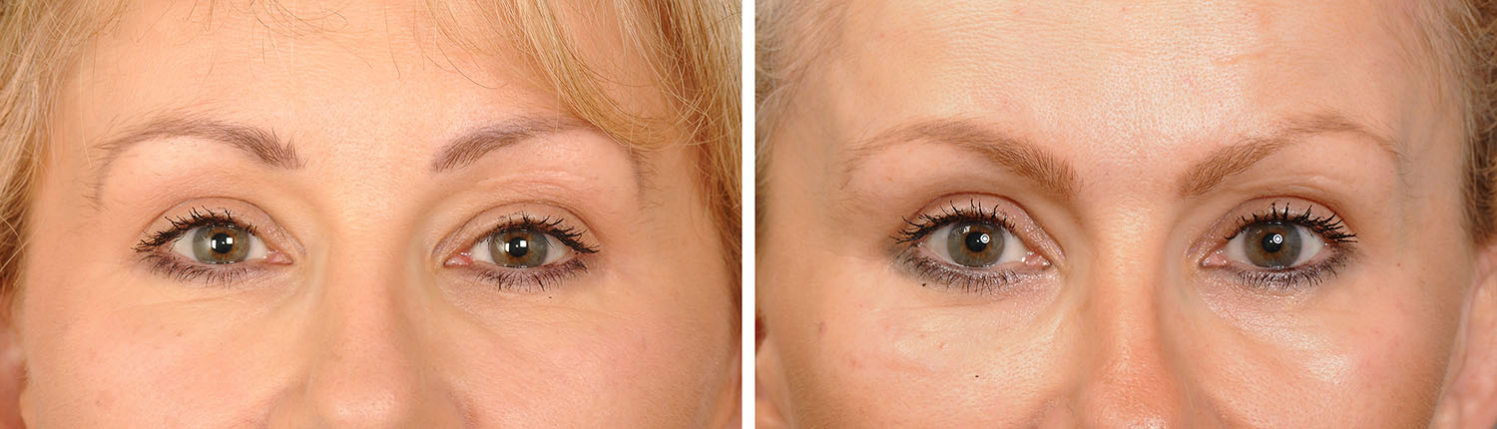
The effect on the ‘eyes’ of improving orbital rim projection alone Left: 45-year-old woman prior to extended lower facelift with midcheek skeletal enhancement. No surgery was performed to her forehead, temples, or upper lids, other than 0.6 mL lipofilling to medial upper lids and 1.2 mL hydroxyapatite augmentation of the superolateral orbital rim. Right. The improved definition of the lateral forehead and brow region helps projection of the apex of the brow

**Fig. 7:**
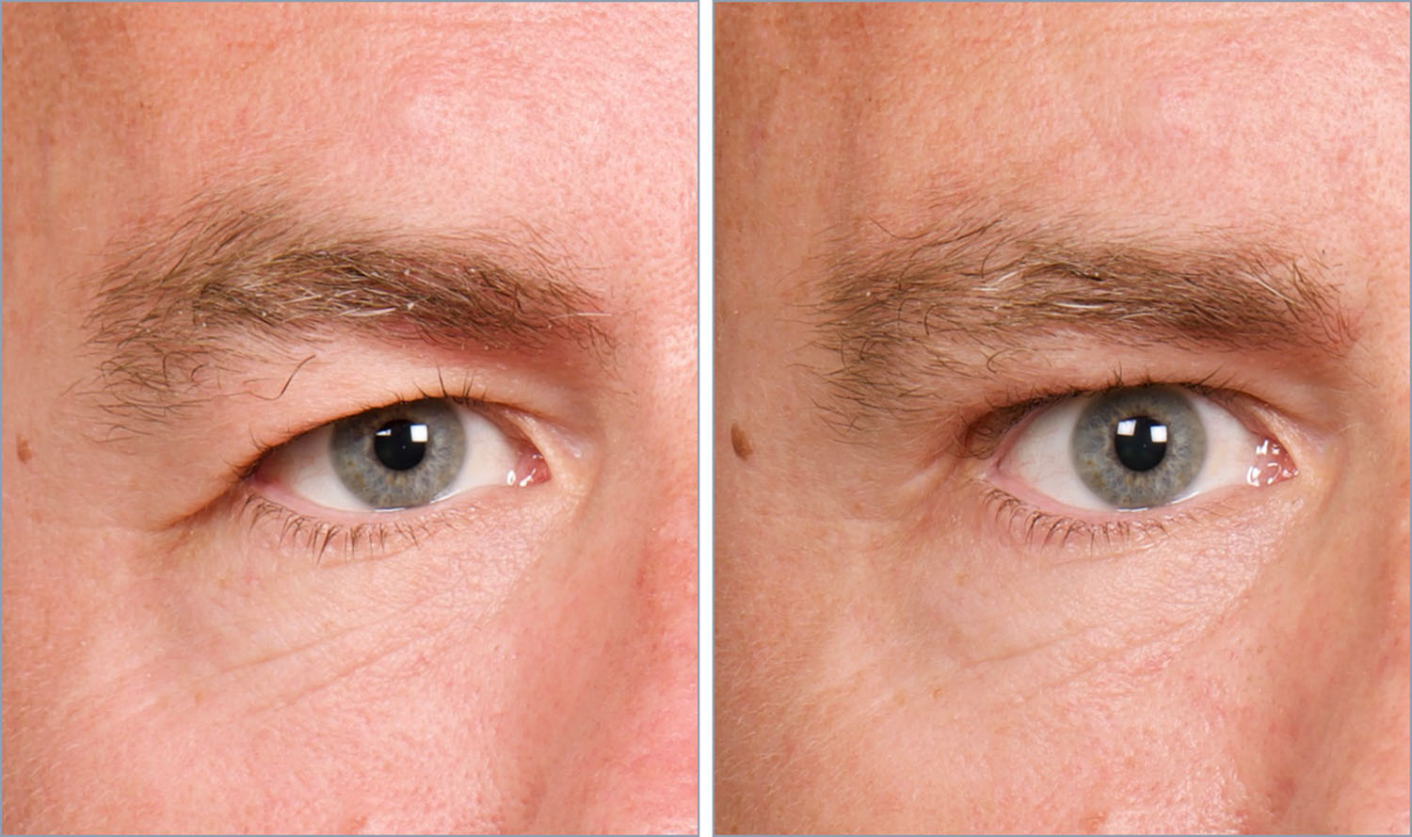
A 50-year-old man, before and one year after 1.4 mL hydroxyapatite augmentation of the superolateral orbital rim and tarsal fixation blepharoplasty. A major reduction of his temporal hooding was obtained while effectively avoiding an ‘operated look’. Observe the slight squaring of his forehead by the ridge of angulation on the lateral boundary of the zygomatic process, strengthening the previously rounded lateral forehead, and adding to his facial attractiveness

**Fig. 8:**
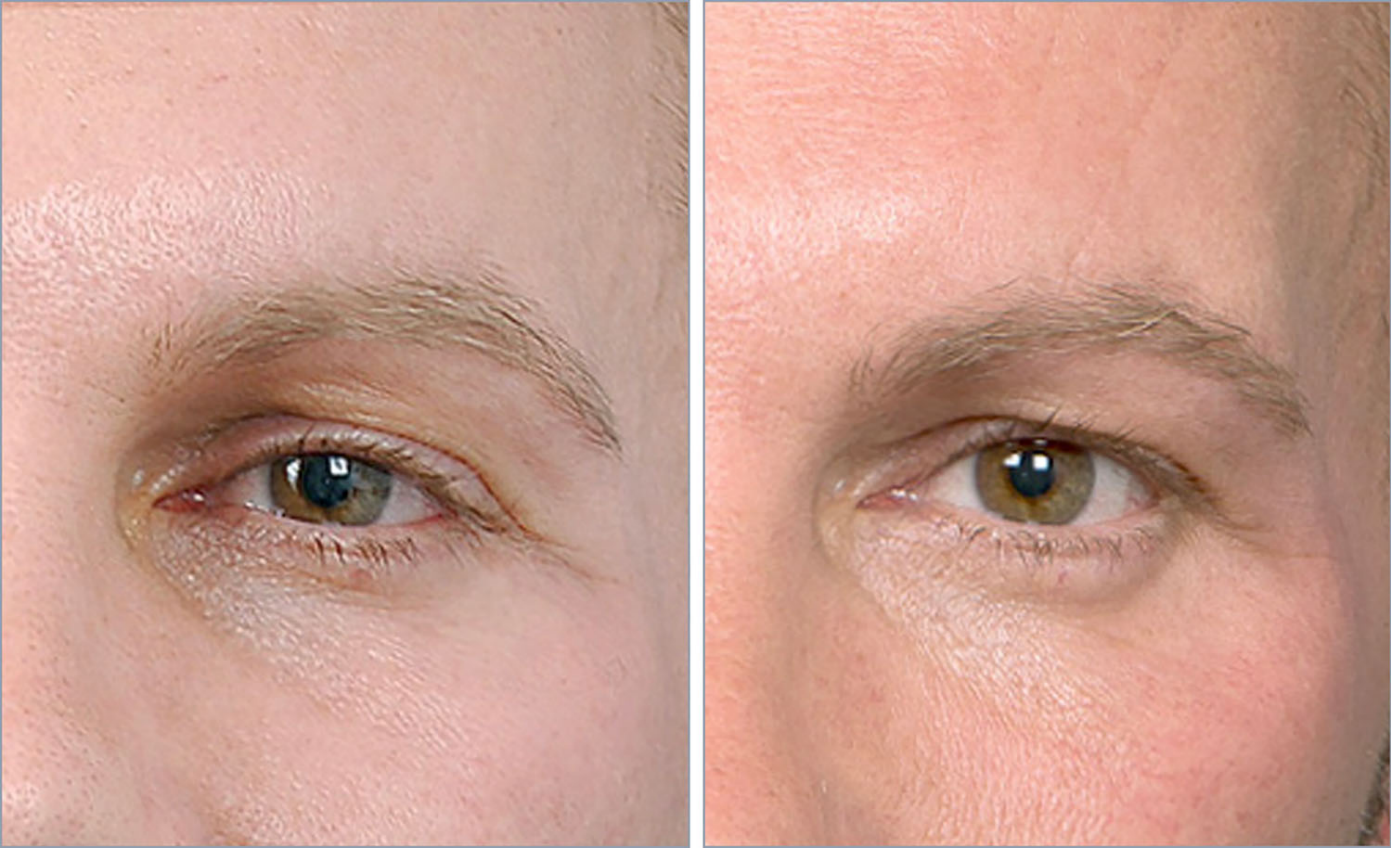
A 44-year-old man before and ten years after 0.6 mL hydroxyapatite augmentation of the superolateral orbital rim. Ancillary procedures included lower facelift with hydroxyapatite augmentation of the maxilla and zygoma. Significantly, the small volume increased projection of the lateral side of the zygomatic process of the frontal bone provides more angulation and contrast between the forehead and a flatter temple. This adds to the masculinity of this patient’s forehead and brow region permanently

**Fig. 9:**
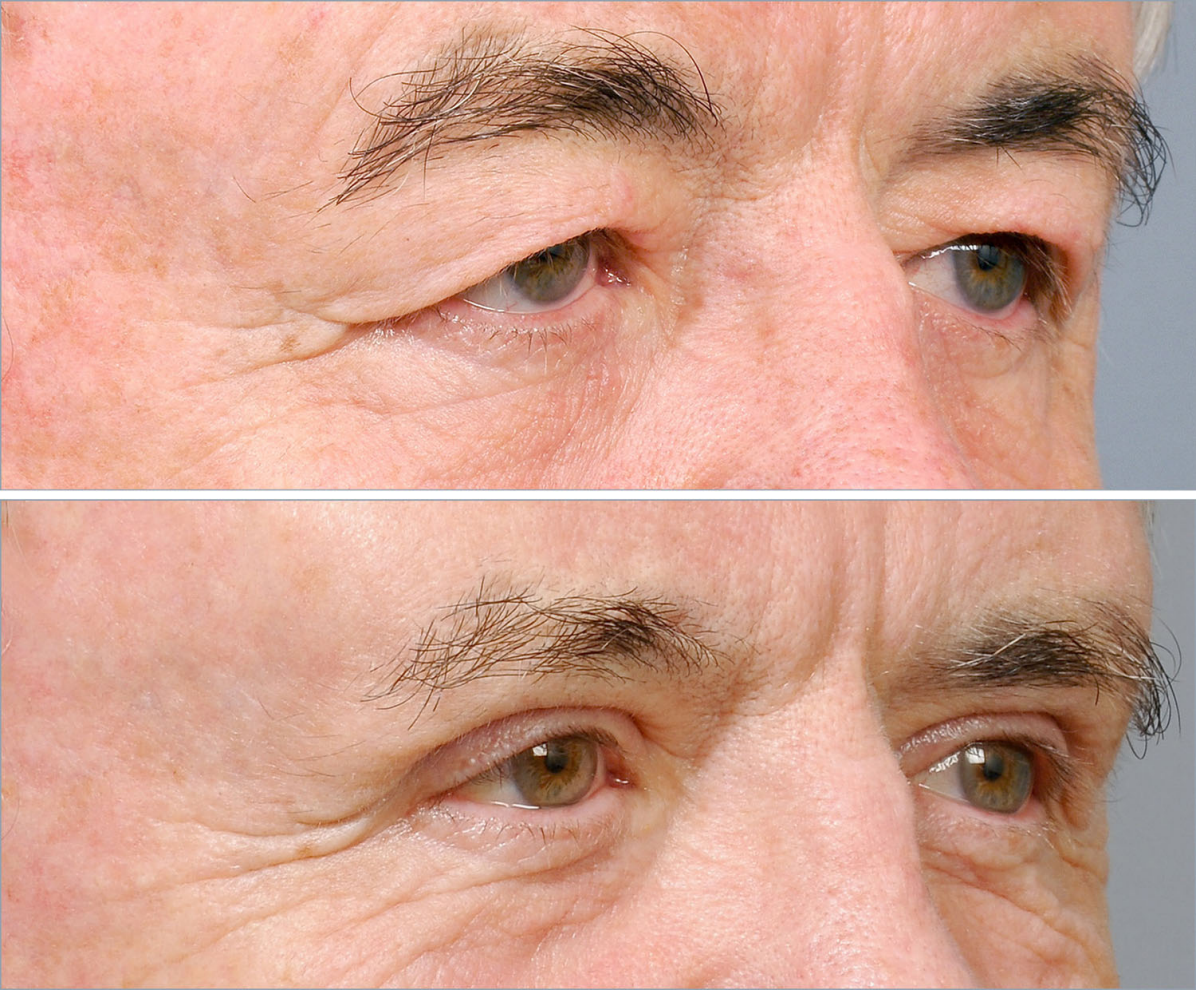
ABOVE. Age 67, preoperative showing prominent temporal hooding of the lid fold superficial to the lateral canthus. BELOW. Age 74, seven years following hydroxyapatite augmentation of the superolateral orbital rim (1.2 mL), along with tarsal fixation blepharoplasty (only 5 mm maximum skin removal) and appropriate contouring of the lid fat pads. The combination of the gentle lift of the lateral brow resulting from the enhanced anterolateral projection of the zygomatic process of the frontal bone, along with tightening of the infrabrow lid skin back into the tarsal crease of the lid leads to a contouring and redraping effect of the lax and ptotic skin that had previously formed the hooding. With a long-term benefit

In 210 patients, (89%), the procedure was effective in enhancing superolateral orbital rim projection (Figs. 5, 6, 7, 8, 9). Additionally, there was a notable increase in infrabrow fullness, with the brow sitting higher in relation to the enhanced orbital rim. This resulted in a higher peaked brow apex and an overall shape improvement.

Twenty-seven patients (11%) subsequently underwent a further procedure for additional volume augmentation of the superolateral orbital rim. Autologous fat injection was used in most of these cases, although additional hydroxyapatite was used in six of the earlier cases.

### Complications

One patient developed a localized unilateral temple hematoma, managed with needle aspiration in the clinic. One patient developed an incision site infection, which responded readily to oral antibiotics, without the need for hydroxyapatite removal. There were not any instances of deep infection involving the implant.

Contour irregularities required correction in 12 patients. Autologous fat injection was used for correction in five and injectable hyaluronic filler in two. One case required a minor surgical revision for correction of a small persistent irregularity due to malposition of some granules. One patient required partial removal of hydroxyapatite on one side only and one patient reported having all the hydroxyapatite removed early postop associated with a facelift at a Thai tourist Clinic. Volume reduction for correction of a small visible bulge, using an injection of Kenacort or Fluorouracil (5-FU), was used in three patients with good results.

One patient had a flare of a pre-existing systemic pain syndrome following hydroxyapatite augmentation in several areas, which including supraorbital. The pain subsided following removal of all hydroxyapatite 6 months after the initial procedure. We did not encounter other complications such as seroma formation and extrusion which have reported incidences of 1.3% and 0.6%, respectively [39].

## Discussion

### Volumizing Procedures

Autologous fat and soft tissue fillers are widely utilized for small volume additions to the brow region [28, 36]. Disadvantages of these modalities are resorption and potential complications including important vascular occlusion [40–42]. Eyelid fat-transposing procedures have recently been described but data on longevity are still lacking [43, 44]. In general, soft tissue augmentation disregards the underlying bone deficiency and thus fail to replace like with like, as espoused by Gillies [45]. This principle was eloquently described by Val Lambros: ‘fat is a filler, while bone is a definer’. Autologous bone grafting has been used in the past, both locally harvested and rib graft [46]. The associated donor-site morbidity and unpredictable high resorption rate of up to 50% detract from its use as an aesthetic surgery adjunct [47]. Solid alloplastic implants such as silicone, expanded polytetrafluoroethylene (sPTFE) and methyl methacrylate have also been widely used, albeit not often for the upper periorbital region [32, 48–50]. The possible use of hydroxyapatite in the superolateral orbital region has been mentioned previously, but technical details were not provided nor were sufficient results reported in long-term follow-up series [30]. Our definitive study is the largest series described for bone augmentation of this region using hydroxyapatite and the first to report on long-term results.

### Hydroxyapatite

Porous hydroxyapatite was introduced in 1974, initially for augmentation of the alveolar ridge. It was later used as an onlay bone-graft substitute [47, 51, 52]. It has proven to be an excellent alloplastic material for improving facial skeletal projection [35, 47, 53]. The unique advantage of porous hydroxyapatite is that it undergoes vascular ingrowth and subsequent incorporation into the host bone [37, 47, 54, 55]. It has the advantage that it does not require fixation, does not initiate a foreign body reaction, does not undergo resorption when placed in the subperiosteal plane, and has a very low chance of infection [30, 37, 39, 47, 48, 56–59]. In this series, the only type of infection was a superficial incisional site infection, which did not require removal of the hydroxyapatite granules.

Commercially available injectable hydroxyapatite mixtures such as Radiesse are not designed for subperiosteal application. Moreover, as this is placed using percutaneous needle injection, it is not possible to place the volume in the subperiosteal plane [60]. The plane of injection would therefore be the preperiosteal fat layer, which does not provide the same longevity as subperiosteal hydroxyapatite granules, because only granules situated directly on the bone surface become incorporated into the bone.

The overall appearance also depends on adequate soft tissue coverage for the supraorbital fullness and for smoothing of the newly formed temporal crest, zygomatic process, and supraorbital rim. Concomitant autologous fat grafting is therefore indicated in cases in the presence of significant soft tissue deflation in the brow region. This was more often the case in older patients in our series. Additionally, the medial supraorbital rim can only be addressed with the lipofilling cannula, as the risk for supraorbital nerve damage deters periosteal elevation in this area.

### Technical Nuances to Improve Outcomes

Early in our series, the most common shortcoming was undercorrection. This is explained by the learning curve for assessment of the volume required, combined with a caution for overcorrection, resulting from not having the benefit of previous guidelines. Following the early experience, there have not been further cases of significant undercorrection since 2011.

The second most common problem was contour irregularities, which also occurred more frequently in the beginning of the series. Contour irregularities are believed to be caused by an iatrogenic slit of the periosteum during dissection allowing migration of some granules into the soft tissues outside the pocket. To limit this complication, we changed our access route. Early in our experience, the usual surgical approach was a remote zig-zag incision within the temporal hair area, to provide natural concealment of the scar. However, in patients having a high hairline, the incision was far from the area of interest, making it difficult to have precision with the subperiosteal pocket dissection and placement of the hydroxyapatite granules. We later appreciated that a small incision in one of the forehead skin creases (marked with the patient raising the brow when still awake) allowed for more precise pocket dissection and ease of delivery of the granules, making the augmentation more accurate, and without the scar being conspicuous. When the subperiosteal pocket remains intact and the inserted granules well contoured, the risk of irregularities is minimal, even in the thin-skinned patient.

### Limitations

A limitation of this study is that it was a retrospective review, acknowledging that a randomized controlled trial comparing fat grafting to hydroxyapatite bone augmentation could be more informative, but not ethical. Another limitation is the recent change to a different source of hydroxyapatite, necessitated by the manufacturer’s discontinuation of the original coralline hydroxyapatite, Pro Osteon 200 for facial use. While similar results are expected with the synthetic coral like replica alternative, long-term outcomes are not yet available. The surgeon’s learning curve, associated with precise and intact pocket dissection and judgement on volume, is a reality, as most of the imperfections (undercorrection, contour irregularities) occurred during the initial years.

## Conclusion

The upper periorbital region is an important, albeit subtle, area in facial aesthetics and attractiveness. Because its significance is not widely appreciated, patients do not present to the surgeon requesting this procedure. The lack of projection in this area is usually overlooked. Surgical correction of this deficiency may therefore need to be suggested by the surgeon. Augmentation of the bone in this region using hydroxyapatite granules is a simple, safe, and predictable procedure that provides permanent aesthetic enhancement without the complexity and risks traditionally associated with alloplastic implants or dermal fillers. The increased projection supports the position of both the brow and the infrabrow lid fold.

In patients lacking aesthetic projection of the superolateral orbital rim, enhancement can be achieved in such an undetectable way that it may resemble the freshness and vitality of youth more aesthetically than can be achieved with traditional lifting procedures alone. This procedure should be considered in the spectrum of upper periorbital aesthetic procedures.

## Supplementary Information

Below is the link to the electronic supplementary material.

Supplementary file 1. Video of the indication setting (MP4 116536 KB)

Supplementary file 2. Video of the procedure (MP4 328729 KB)
