# Correction: Aesthetic Enhancement of the Brow using Hydroxyapatite

**DOI:** 10.1007/s00266-022-02890-y

**Published:** 2022-04-28

**Authors:** Lennert Minelli, Jacqueline Richa, Bryan C. Mendelson

**Affiliations:** 1 Melbourne Advanced Facial Anatomy Course (MAFAC), 109 Mathoura Road Toorak, Melbourne, VIC 3142 Australia; 2 The Panama Clinic, Calle Ramón H Jurado, Centro Pacific Center Torre B, Panama, Panama; 3 The Centre for Facial Plastic Surgery, 109 Mathoura Road Toorak, Melbourne, VIC 3142 Australia

## Correction to: Aesth Plast Surg 10.1007/s00266-022-02793-y

This article was updated to remove black boxes introduced to Fig. 2 after the author reviewed proofs and to change the placement of the quoted text.

